# High dietary *Mucuna pruriens utilis* seed meal compromises growth performance, carcass traits, haemato-biochemistry, and meat quality of broilers

**DOI:** 10.1007/s11250-024-04120-w

**Published:** 2024-10-01

**Authors:** Makiwa Simeon Mthana, Doctor Mziwenkosi Nhlanhla Mthiyane

**Affiliations:** 1 Department of Animal Science, School of Agricultural Sciences, Faculty of Natural and Agricultural Sciences, North-West University (Mahikeng Campus), Private Bag X 2046, Mmabatho, 2735 South Africa; 2 Food Security and Safety Focus Area, Faculty of Natural and Agricultural Sciences, North-West University (Mahikeng Campus), Mmabatho, 2735 South Africa

**Keywords:** Broilers, Performance, Haemato-biochemistry, Meat quality, *Mucuna pruriens utilis*

## Abstract

Usage of soyabean meal (SBM) in broiler diets is economically and environmentally unsustainable thus necessitating investigation of alternative protein sources. Therefore, this study investigated effects of incremental inclusion levels of *Mucuna pruriens utilis* seed meal (MSM) for partial substitution of SBM in broiler diets. In a completely randomized design (CRD), 400 day-old Ross 308 chicks were allotted to 5 iso-caloric-nitrogenous MSM-containing (0, 5, 10, 15, and 20%) dietary treatments. Each treatment was replicated 8 times, with each pen having 10 birds, during starter (d1 – 14), grower (d15 – 28), and finisher (d29 – 42) phases. Results showed that dietary MSM decreased feed intake (FI: quadratic: *P* < 0.001), body weight gain (BWG: linear:* P* < 0.001), and feed conversion efficiency (FCE: linear: *P* < 0.001) as it linearly decreased slaughter weight (SW: *P* < 0.001), hot carcass weight (HCW: *P* < 0.001), cold carcass weight (CCW: *P* < 0.001), dressing percentage (*P* < 0.001), and breast weight (*P* < 0.05). In contrast, dietary MSM linearly increased the weights of the liver (*P* < 0.01), proventriculus (*P* < 0.001), gizzard (*P* < 0.001), duodenum (*P* = 0.01), jejunum (*P* < 0.001), ileum (*P* < 0.001), caecum (*P* < 0.01), and colon (*P* < 0.01). Also, dietary MSM quadratically increased blood heterophils (*P* < 0.05) and alkaline phosphatase activity (*P* < 0.05) of the chickens whilst linearly increasing their serum amylase (*P* = 0.001) and lipase (*P* = 0.001) activities and linearly decreasing their serum symmetric dimethylarginine (SDMA: *P* = 0.001) and cholesterol (*P* < 0.05). Further, dietary MSM linearly decreased chicken breast meat ultimate pH (*P* < 0.05) whilst linearly increasing its cooking loss (*P* < 0.01), drip loss (*P* < 0.05) and shear force (*P* < 0.01). In conclusion, dietary MSM compromised growth performance, carcass characteristics, and meat quality of broilers as it increased the weights of their digestive-metabolic organs.

## Introduction

Broiler production has exponentially increased over the past decade thus creating optimism in terms of satisfying the ever-growing demand for food as the world human population burgeons particularly in Africa to reach 10.4 billion in 2100 (Haque et al. 2020; UN 2023). Also, due to its relative affordability and less capital and minimal land requirements for its production, among other things, broiler meat continually experiences a huge demand worldwide (Dörper et al. 2021). Notwithstanding, resource-limited (smallholder) farmers mainly in developing countries experience poor levels of productivity and low offtake (Otiang et al. 2020) due to high purchasing price of broiler feeds accounting for approximately 70% of the total production costs (Salami et al. 2019; Mallick et al. 2020; Li et al. 2021). This emanates mainly from predominant utilization of the expensive soyabean meal (SBM) as a protein-supplying ingredient when formulating broiler diets. Also, global large-scale production of soyabean negatively impacts the environment due to land deforestation and degradation, depletion of fossil fuels, emission of gases that pollute the atmosphere and induce global warming, acidification and eutrophication of water bodies, and perturbation of faunal and floral biodiversity (Pexas et al. 2023). Further, its production requires huge financial investments in the form of fossil fuels, labour, land and synthetic and chemical inputs (FAO 2016). Consequently, this significantly raises the costs of importing and transporting soyabeans over long distances (Taghizadeh-Hesary et al. 2019), with smallholder poultry farmers bearing the brunt of the associated financial risks. Furthermore, soyabean use in the nutrition of broilers and livestock production operations increases the stiffness of the feed-food competition between animals and humans (Breewood and Garnett 2020). For these reasons, there is a need for investigation of cheaper, sustainable, and nutritionally adequate alternative protein sources for broiler diets the production of which circumvents the above-mentioned limitations and use of which as dietary ingredient enhances the quality and healthiness of meat.

The seed meal (MSM) of *Mucuna pruriens* (L.) DC var. *utilis* (Wall. ex Wight) Baker ex Burck (velvet bean) is a promising alternative protein source with a relatively high content of crude protein (CP: 21 – 38%) (Fathima et al. 2010) and amino acids methionine and lysine (> 6%) (Tuleun et al. 2009) comparably to SBM (Banaszkiewicz 2011). Also, MSM contains bioactive compounds including tannins, flavonoids, and phenolic compounds, low concentrations of which have notable antioxidant, antidiabetic / hypoglycaemic (Manhivi et al. 2022; Mashau et al. 2022), hypolipidemic, and low-density lipoprotein cholesterol lowering effects in animals (Jayaweera et al. 2007; Ekpo et al. 2008; Ngatchic et al. 2016). These bioactive constituents may also improve the immune system and thus healthiness of broilers while enhancing the quality of their meat for human consumption (Ndudzo et al. 2023). Notwithstanding, the greatest challenge limiting widespread utilization of MSM particularly in broiler nutrition is its high content (≤ 11%) of the potentially toxic amino acid 3,4-dihydroxy-L-phenylalanine (L-DOPA) (Pulikkalpura et al. 2015; Konishi et al. 2022).

Consequently, consumption of MSM-supplemented diets decreases body weight gain (BWG), feed intake (FI) and feed conversion efficiency (FCE) whilst increasing the weights of essential metabolic-digestive organs such as the liver, pancreas and intestines in broilers particularly when included at high levels (≥ 15%) (Del Carmen et al. 1999; Iyayi et al. 2006; Vadivel and Pugalenthi 2010). On the other hand, as a precursor for catecholamines, MSM-derived L-DOPA has muscle accretion enhancing properties with ability to increase carcass (meat) yields (Navegantes et al. 2000; Alleman et al. 2011; Lieu et al. 2012). However, this aspect of MSM has not been investigated in prior studies. Also, the effect of feeding diets supplemented with high levels of MSM on the whole kaleidoscope of haematological and serum biochemistry parameters to reveal its possible influence on the health and nutritional status of broilers from starter to finisher phases has hitherto not been investigated. Such information is imperative in determining the optimum inclusion level of MSM in broiler diets. Therefore, the objective of this study was to determine the effects of dietary supplementation with incremental inclusion levels of MSM on productive performance, carcass traits, visceral organs, haemato-biochemistry, and meat quality of broilers.

## Materials and methods

### Sourcing of materials and chemical analysis

Ross 308 broiler chicks were purchased from Superior Chicks (Pty) Ltd. (Pretoria, South Africa) and transported to North-West University (NWU) Molelwane Experimental farm in Ngaka Modiri Molema District (GPS: 25°40.459′S, 26°10.563′E) under Mahikeng Local Municipality (North West Province, South Africa) where the study was conducted. *M. pruriens utilis* seeds were sourced from AGT Foods Pty (Ltd) (Krugersdorp, South Africa) whereas all other dietary ingredients were from SimpleGrow Agric Services (Pty) Ltd. (Centurion, Gauteng Province, South Africa). The seeds were then milled (screen size: 1 mm) and chemically analysed alongside the experimental diets (Tables 1 and 2). Dry matter (DM) (method 930.15), crude protein (CP) (method 954.01), crude fibre (Method 930.10), ether extract (EE) (method 920.39), ash / organic matter (OM) (method 942.05), and amino acids (method 985.28) were analysed using AOAC (2005) methods whilst neutral detergent fibre (NDF), acid detergent fibre (ADF) and acid detergent lignin (ADL) were analysed following procedures of Van Soest et al. (1991). Then digestible energy (DE) was calculated using the formula: DE (MJ/kg) = 17.47 + (0.0076 × CP g/kg) + (0.0158 × EE g/kg)—(0.0331 × ash g/kg)—(0.0140 × NDF g/kg) (McDonald et al. 2010). Also, metabolizable energy (ME) was calculated as ME (kcal/kg) = 2778 – 66 (CF × 88%DM) (Livesey 1989) and converted into MJ/kg.

**Table 1:** Nutritional composition (g/kg DM, unless otherwise stated) of *M. pruriens utilis* seed meal

Parameter	Amount
*Proximate (g/kg)*	
Dry matter	912.7
Crude protein	232.7
Ether extract	84.5
Ash	34.2
Organic matter	965.8
Neutral detergent fibre	368.6
Acid detergent fibre	146.2
Acid detergent lignin	71.8
Digestible energy (MJ/kg)	14.27
*Amino acids (g/100 g protein)*	
Alanine	2.24
Glycine	3.64
Valine	2.66
Leucine	4.37
Isoleucine	2.42
Proline	5.10
Methionine	0.44
Serine	2.81
Threonine	3.86
Phenylalanine	2.21
Aspartic acid	8.14
Cysteine	1.46
Glutamic acid	6.98
Asparagine	0.13
Lysine	8.75
Glutamine	0.42
Histidine	2.11
Tyrosine	2.46
Cystine	0.62

**Table 2:** Composition of experimental diets (g/kg, unless stated otherwise)

Item	Starter (d1—14)					Grower (d15—28)					Finisher (d29—42)				
	0	5	10	15	20	0	5	10	15	20	0	5	10	15	20
Yellow maize	587.7	582.3	557.1	507.7	479.8	600.1	591.7	588.0	561.0	466.6	604.5	587.2	583.3	579.7	511.2
Soyabean full fat (42%)	124.9	44.5	0	0	0	150.0	126.2	42.5	1.5	70.0	150.0	233.6	150.2	66.4	98.4
SBM (46.5% CP)	255.0	290.6	270.7	183.5	132.1	211.9	202.6	238.6	210.5	50.1	190.4	100.4	136.4	172.4	42.1
Sunflower oilcake (34% CP)	0	0	38.3	122.8	150.0	0	0	0	44.1	178.1	0	0	0	0	114.1
MSM (23% CP)	0	50.0	100.0	150.0	200.0	0	50.0	100.0	150.0	200.0	0	50.0	100.0	150.0	200.0
Feed lime fine	13.9	13.6	13.3	12.9	12.7	12.8	12.5	12.3	12.0	11.4	13.1	12.9	12.7	12.5	12.0
Mono-dicalcium phosphate	9.9	10.3	10.7	10	11.2	7.3	7.8	8.2	8.6	8.7	7.7	8.2	8.7	9.1	9.3
BSGF Premix*	3.0	3.0	3.0	3.0	3.0	2.5	2.5	2.5	2.5	2.5	2.0	2.0	2.0	2.0	2.0
Salt fine	2.8	2.7	2.7	2.7	2.2	2.8	2.7	2.7	2.6	2.0	2.8	2.8	2.7	2.6	2.4
Sodium bicarbonate	1.0	1.0	1.0	1.0	1.4	1.0	1.0	1.0	1.0	1.7	1.0	1.0	1.0	1.0	1.2
Soyabean oil crude	0	0	0	0	0	9.7	0	0	0	0	27.0	0	0	0	0
L-Lysine	0	0	1.0	2.7	3.9	0	0.7	1.4	2.7	4.6	0	0.1	0.8	1.6	3.6
DL-Methionine	1.1	1.4	1.6	1.8	2.1	1.3	1.7	2.0	2.3	2.2	0.8	1.2	1.5	1.8	2.0
L-Threonine	0	0	0	0.3	0.7	0	0	0.1	0.6	1.0	0	0	0	0.2	0.7
L-Valine	0	0	0	0	0.2	0	0	0	0.1	0.4	0	0	0	0	0.2
Choline chloride	0.3	0.3	0.3	0.3	0.3	0.3	0.3	0.3	0.3	0.3	0.3	0.3	0.3	0.3	0.3
Betaine	0.3	0.3	0.3	0.3	0.3	0.3	0.3	0.3	0.3	0.3	0.3	0.3	0.3	0.3	0.3
Quantum blue 10000G	0.1	0.1	0.1	0.1	0.1	0.1	0.1	0.1	0.1	0.1	0.1	0.1	0.1	0.1	0.1
Total	1000	1000	1000	1000	1000	1000	1000	1000	1000	1000	1000	1000	1000	1000	1000
Chemical composition															
Dry matter	879.6	881.5	884.5	888.8	892.4	880.2	881.9	883.9	887.1	893.2	881.9	883.0	885.0	887.0	892.1
Crude protein	210.0	210.0	210.0	210.0	210.0	200.0	200.0	200.0	200.0	200.0	190.0	190.0	190.0	190.0	190.0
Ash	42.2	42.6	43.5	45.0	46.1	38.4	38.7	39.0	40.0	42.6	37.4	37.9	38.2	38.5	40.5
Ether extract	49.9	41.6	39.6	45.3	51.2	64.2	56.7	47.9	46.5	63.8	81.0	76.0	67.3	58.5	69.7
Crude fibre	28.7	28.9	35.5	49.8	58.0	28.8	30.5	30.5	38.0	61.5	28.0	32.7	32.8	32.8	52.3
Metabolizable energy (MJ/Kg)	11.5	11.5	11.5	11.5	11.5	12.0	12.0	12.0	12.0	12.0	12.5	12.5	12.5	12.5	12.5
Calcium	10.0	10.0	10.0	10.0	10.0	9.0	9.0	9.0	9.0	9.0	9.0	9.0	9.0	9.0	9.0
Total phosphorus	5.9	6.0	6.2	6.6	6.9	5.2	5.3	5.4	5.6	6.2	5.1	5.2	5.3	5.4	5.9
Potassium	9.8	9.9	10.0	9.9	10.0	9.3	9.4	9.5	9.5	9.5	8.8	8.9	9.0	9.1	9.0
Sodium	1.6	1.6	1.6	1.6	1.6	1.6	1.6	1.6	1.6	1.6	1.6	1.6	1.6	1.6	1.6
Chloride	2.2	2.1	2.2	2.6	2.6	2.2	2.2	2.3	2.5	2.6	2.2	2.1	2.2	2.3	2.6

*MSM* mucuna seed meal; *SBM* soyabean meal

*BSGF Premix: 0.12 g biotin; 0.7 mg folic acid; 30 mg niacin; 2.5 mg vitamin B1; 10 mg pantothenic acid; 11,000 IU vitamin A; 4.5 mg vitamin B2; 1 mg vitamin B6; 2.0 mg vitamin K3; 2500 IU vitamin D3; 25 IU vitamin E; 100 mg magnesium sulphate; 8.0 mg copper sulphate; 0.25 mg sodium selenite; 80 mg ferrous sulphate; 79 mg zinc sulphate; 0.34 mg potassium iodine.

### Diet formulation, study design, and bird management

Experimental procedures employed in the execution of this study were approved by the Animal Production Sciences Research Ethics Committee of NWU (Ethics Number: NWU-00806–22-A5). Five dietary treatments containing varying inclusion levels (0, 5, 10, 15, and 20%) of MSM replacing SBM were formulated to be iso-energetic and iso-nitrogenous to meet the nutritional needs of broilers at different growth phases [starter (day 1 – 14), grower (day 15 – 28), and finisher (day 29 – 42)] (Table 2). The diets were then assigned to 400 day-old Ross 308 chicks (initial weight 39.98 ± 0.592 g). Each treatment was replicated 8 times with each replicate pen having 10 chicks (5 males: 5 females) in a completely randomised design (CRD). Each replicate pen was treated as an experimental unit. On chick placement, the birds were given vitamins and electrolytes (Stresspac, Phenix®) through drinking water for the first 48 h to manage stress induced by transportation. The birds were reared for 42 days in an environmentally controlled broiler house with temperature maintained between 25 and 30 ℃, artificial lighting provided for 24 h a day, and ventilation controlled by opening the semi-automatic curtains during the day. Each pen had an infra-red lamp for brooding during the first week. The birds had free access to fresh water and feed throughout the experimental trial.

### Growth performance

The chicks were group-weighed per pen at placement and again at weekly intervals to obtain weekly body weights, which were used to calculate the BWG by subtracting the previous body weight from the current body weight and divided by the number of birds per pen. Feed intake (FI) per bird was measured daily by subtracting the feed refusals from the feed offered and then dividing by number of birds per pen. Then the weekly FI was calculated by summing the daily measured FI over 7 days. The FCE was calculated by dividing BWG with FI.

### Haemato-biochemistry

Whole blood was collected from 2 birds per pen from the wing vein using a 21-gauge needle. Approximately 1 mL of blood samples were transferred into ethylenediaminetetraacetic acid (EDTA)-coated vacutainer tubes for haematological analysis using a computerized IDEXX LaserCyte Haematology Analyzer (IDEXX Laboratories (Pty) Ltd., Johannesburg, South Africa). On the other hand, approximately 4 mL of fresh blood samples were transferred into Vacuette® Serum Clot Activator tubes, centrifuged (10000 rmp for 10 min), and the serum analysed for biochemical parameters using a computerized IDEXX Vet Test Chemistry Analyzer (IDEXX Laboratories (Pty) Ltd., Johannesburg, South Africa).

### Slaughter procedure and carcass traits

On the day of slaughter, 6 birds per pen (3 males: 3 females) were randomly selected, group-weighed and transported to a commercial Rooigrond poultry abattoir for slaughter. The birds were humanely slaughtered by cervical dislocation after electrical stunning using a sharp knife and allowed to thoroughly bleed. They were defeathered and the heads, necks, and feet removed. Thereafter, they were eviscerated by removing the visceral organs through an opening from the vent. The organs were then weighed individually and expressed relative to slaughter weight. Hot carcass weights (HCW) were recorded instantly after slaughter, then the carcasses were chilled (4 ℃ for 24 h), and the cold carcass weights (CCW) recorded. The left breast, thigh, drumstick, and wing were separated by cutting from the joints of the carcass. All the carcass cuts were weighed and expressed relative to their CCW.

### Meat quality analysis

All meat quality parameters were measured on the breast meat. Meat pH was measured using a specialised meat pH meter (Hanna Instruments, HI98163, Romania) while colour coordinates [lightness (L*), redness (a*), and yellowness (b*) were measured from 3 different meat surface locations using a Konica Minolta colour guide [Narich (Pty) Ltd, Cape Town, RSA]. Metric Hue-angle (H*) and Chroma (C*) values were determined as described by the American Meat Science Association (AMSA 2012). Water holding capacity (WHC) and drip loss were determined as described by Honikel (1998). Cooking loss was measured by cooking the pre-weighed meat samples at 85 ℃ for 45 min (Ding et al. 2010). After cooking and cooling, the meat was cored parallel to the muscle fibre direction and sheared perpendicular to their direction using a Meullenet-Owens’s razor shear blade mounted on a Texture Analyser (Stable Micro System, Model: TA-XT plus, Surrey GU7 1YL, UK).

### Statistical analysis

Repeated measures analysis was performed on the weekly measured data (FI, BW, BWG, and FCE) using the Generalised Linear Model (GLM) procedure of SAS (2012) with the statistical model:

$${Y}_{ijk}=\mu +{\alpha }_{i}+{\beta }_{j}+{\left(\alpha \beta \right)}_{ij}+{\varepsilon }_{ijk}$$

where Y_*ijk*_ is the dependant variable, µ is the population mean, α_*i*_ is the effect of diet, β_*j*_ is the effect of week, (αβ)_*ij*_ is the diet × week interaction effect, and Ɛ_ijk_ is the random error. The rest of the data were subjected to One-way ANOVA using the PROC GLM procedure with the statistical model:

$${Y}_{ij}=\mu +{\alpha }_{i}+{\varepsilon }_{ij}$$

where Y_*ijk*_ is the dependant variable, µ is the population mean, α_*i*_ is the effect of diet, and Ɛ_*ijk*_ is the random error. The probability of difference (PDIFF) option was used to separate the Least Square Means. The differences between means were considered when *P* ≤ 0.05. Thereafter, linear and quadratic responses to dietary MSM were assessed using response surface regression (Proc RSREG) analysis. The optimum response of MSM was estimated using the quadratic model:

$$y={ax}^{2}+bx+c$$

where y is the dependant variable; a and b are the coefficients of the quadratic equation; c is the intercept; *x* is the MSC level (%); and − b/2a = x value is for determining the optimal response of MSC.

## Results

### Growth performance

The effect of dietary inclusion of MSM on weekly and overall FI, BW, BWG, and FCE of broilers is shown in Table 3, with their respective regression equations in Table 4. Repeated measures analysis showed a diet × week interaction effect on weekly FI (*P* < 0.001), BW (*P* < 0.001), BWG (*P* < 0.001), and FCE (*P* < 0.001). Dietary inclusion of MSM quadratically decreased FI in weeks 1 (*P* < 0.001), 2 (*P* < 0.001), 3 (*P* < 0.001), 4 (*P* < 0.001), 5 (*P* < 0.001), and 6 (*P* < 0.05). Overall, FI was also quadratically decreased (*P* < 0.001) by increasing levels of MSM, with the optimum dietary inclusion level of the seed meal at 19% for this parameter. Also, dietary MSM linearly decreased BW in weeks 1 (*P* < 0.01), 2 (*P* < 0.001), 4 (*P* < 0.001), 5 (*P* < 0.001), and 6 (*P* < 0.001) as it quadratically decreased this parameter in week 3 (*P* < 0.001). Overall, BW was also decreased linearly (*P* < 0.001) by dietary MSM. Regarding BWG, dietary MSM linearly decreased this parameter in weeks 1 (*P* < 0.01), 2 (*P* < 0.001), 4 (*P* < 0.001), 5 (*P* < 0.001), and 6 (*P* < 0.001) as it quadratically decreased the parameter in week 3 (*P* < 0.001). Overall, dietary MSM linearly decreased BWG (*P* < 0.001). In terms of FCE, MSM linearly decreased this parameter in weeks 1 (*P* < 0.001), 2 (*P* < 0.001), 3 (*P* < 0.05), 4 (*P* < 0.001), 5 (*P* < 0.001), and 6 (*P* < 0.001). Similarly, overall FCE was also linearly decreased (*P* < 0.001) by dietary MSM.

**Table 3:** Effect of dietary inclusion of MSM on weekly and overall productive performance of broilers

Parameter	Week	Dietary inclusion of MSM (%)					SEM	*P*-value				
		0	5	10	15	20		Linear	Quadratic	Diet	Week	Diet × Week
FI (g/bird)	1	82.95^b^	83.79^b^	87.79^ab^	93.66^a^	70.49^c^	2.8	0.148	0.001	*P* < 0.001	*P* < 0.001	*P* < 0.001
	2	162.26^b^	160.97^b^	179.75^a^	191.11^a^	113.54^c^	9.6	0.061	0.001			
	3	333.40^b^	335.45^b^	372.42^a^	397.42^a^	251.44^c^	14.2	0.075	0.001			
	4	431.68^c^	497.33^b^	573.09^a^	606.70^a^	405.12^c^	13.6	0.344	0.001			
	5	763.89^b^	789.44^b^	806.98^ab^	835.46^a^	686.41^c^	15.7	0.065	0.001			
	6	992.70^b^	1010.99^ab^	1041.55^ab^	1053.99^a^	1012.16^ab^	17.1	0.136	0.036			
BW (g/bird)	1	95.26^a^ 193.01^a^ 318.49^b^ 653.59^a^ 1219.50^a^ 1902.19^a^	91.18^a^ 182.52^a^ 317.51^b^ 675.57^a^ 1185.53^a^ 1770.94^ab^	94.97^a^ 194.09^a^ 438.89^a^ 749.02^a^ 1170.74^a^ 1711.88^bc^	98.68^a^ 198.29^a^ 407.54^a^ 677.15^a^ 1099.99^a^ 1559.06^c^	78.72^b^ 131.57^b^ 269.41^b^ 447.99^b^ 747.16^b^ 1171.25^d^	2.16 7.42 13.01 25.43 30.84 40.96	0.005 0.0004 0.889 < 0.0001 < 0.0001 < 0.0001	0.003 0.0007 < 0.0001 < 0.0001 < 0.0001 0.001	*P* < 0.001	*P* < 0.001	*P* < 0.001
	2											
	3											
	4											
	5											
	6											
BWG (g/bird)	1	55.62^ab^	51.15^b^	55.35^ab^	57.67^a^	39.11^c^	2.1	0.003	0.004	*P* < 0.001	*P* < 0.001	*P* < 0.001
	2	97.75^a^	91.34^a^	99.12^a^	99.61^a^	52.84^b^	6.3	0.001	0.002			
	3	125.49^b^	134.99^b^	244.80^a^	209.24^a^	137.84^b^	13.9	0.070	0.001			
	4	335.09^a^	358.06^a^	310.13^a^	269.61^b^	178.59^c^	29.3	0.001	0.047			
	5	565.91^a^	509.96^ab^	421.72^b^	422.84^b^	299.16^c^	36.2	0.001	0.735			
	6	682.69^a^	585.41^b^	541.13^bc^	459.07^c^	424.09^c^	32.4	0.001	0.469			
FCE	1	0.67^a^	0.61^b^	0.63^ab^	0.62^b^	0.56^b^	0.02	0.001	0.687	*P* < 0.001	*P* < 0.01	*P* < 0.001
	2	0.60^a^	0.57^a^	0.55^ab^	0.52^b^	0.46^c^	0.02	0.001	0.311			
	3	0.55^a^	0.53^ab^	0.67^a^	0.42^b^	0.39^b^	0.05	0.018	0.055			
	4	0.66^a^	0.68^a^	0.54^ab^	0.45^b^	0.44^b^	0.06	0.001	0.984			
	5	0.74^a^	0.64^ab^	0.52^b^	0.51^b^	0.44^b^	0.04	0.001	0.330			
	6	0.69^a^	0.58^b^	0.52^bc^	0.44^c^	0.42^c^	0.04	0.001	0.218			
IBW (g/bird) Overall FI (g/bird)		39.65 2852.91^b^	39.62 2909.63^b^	41.01 3061.59^ab^	39.62 3178.35^a^	40.03 2539.16^c^	0.59 54.4	0.635 0.113	0.443 0.001			
Overall BW		1902.19^a^	1770.94^ab^	1711.88^bc^	1559.06^c^	1171.25^d^	40.96	< 0.0001	0.001			
Overall BWG (g/bird)		1862.54^a^	1730.91^b^	1672.26^b^	1518.05^c^	1131.64^d^	41.1	0.001	0.001			
Overall FCE		0.65^a^	0.60^b^	0.55^c^	0.48^d^	0.45^d^	0.01	0.001	0.515			

^a,b,c,d^ Means within the same row having different superscripts are considered significantly different

*BW* Body weight; *BWG* body weight gain; *FCE* feed conversion efficiency; *FI* feed intake; *IBW* Initial body weight; *MSM* mucuna seed meal; and *SEM* standard error of the mean

**Table 4:** Regression equations of weekly and overall productive performance

Parameter	Week	Regression equations	R^2^	*P*-value
FI	1	y = -0.13(± 0.034)x^2^ + 2.34(± 0.717)x + 80.15(± 3.028)	0.273	*P* < 0.001
	2	y = -0.46(± 0.117)x^2^ + 7.80(± 2.456)x + 152.13(± 10.366)	0.270	*P* < 0.001
	3	y = -0.88(± 0.188)x^2^ + 15.56(± 3.921)x + 314.41(± 16.551)	0.3519	*P* < 0.001
	4	y = -1.65(± 0.199)x^2^ + 34.07(± 4.141)x + 409.16(± 17.480)	0.6448	*P* < 0.001
	5	y = -0.97(± 0.194)x^2^ + 17.15(± 4.040)x + 749.91(± 17.054)	0.3800	*P* < 0.001
	6	y = -0.40(+ 0.182)x^2^ + 9.55(± 3.791)x + 986.13(± 16.003)	0.1074	*P* < 0.05
BW	1	y = -0.09(± 0.029)x^2^ + 1.31(± 0.598)x + 92.33(± 2.523)	0.1792	*P* < 0.01
	2	y = -0.34(± 0.093)x^2^ + 4.71(± 1.943)x + 184.19(± 8.202)	0.2058	*P* < 0.001
	3	y = -1.22(± 0.198)x^2^ + 24.24(± 4.122)x + 290.99(± 17.399)	0.5072	*P* < 0.001
	4	y = -1.85(± 0.291)x^2^ + 28.81(± 6.077) + 630.07(± 25.652)	0.4036	*P* < 0.001
	5	y = -1.98(± 0.363)x + 1191.53(± 31.969)	0.5793	*P* < 0.001
	6	y = -1.73(± 0.459)x + 1871.12(± 40.393)	0.7478	*P* < 0.001
BWG	1	y = -0.09(± 0.028)x^2^ + 1.19(± 0.578)x + 52.78(± 2.440)	0.1685	*P* < 0.01
	2	y = -0.25(± 0.073)x^2^ + 3.40(± 1.537)x + 91.86(± 6.489)	0.1859	*P* < 0.01
	3	y = -0.88(± 0.180)x^2^ + 19.53(± 3.744)x + 106.79(± 15.804)	0.3713	*P* < 0.001
	4	y = -0.63(± 0.306)x^2^ + 4.57(± 6.388)x + 339.08(± 26.962)	0.0695	*P* < 0.05
	5	y = -9.78(± 8.057)x + 561.46(± 34.008)	0.4428	*P* < 0.001
	6	y = -17.83(± 7.064)x + 679.584(± 29.818)	0.5236	*P* < 0.001
FCE	1	y = -0.003(± 0.004)x + 0.65(± 0.017)	0.2652	*P* < 0.001
	2	y = -0.002(± 0.004)x + 0.59(± 0.018)	0.4095	*P* < 0.001
	3	y = -0.03(± 0.012)x + 0.37(± 0.052)	0.1300	*P* < 0.05
	4	y = -0.01(± 0.012)x ± 0.69(± 0.052)	0.2861	*P* < 0.001
	5	y = -0.02(± 0.009)x + 0.74(± 0.039)	0.4552	*P* < 0.001
	6	y = -0.02(± 0.008)x + 0.69(± 0.033)	0.5037	*P* < 0.001
Overall FI		y = -4.08(± 0.748)x^2^ + 74.369(± 15.602)x + 2 776.22(± 65.856)	0.4284	*P* < 0.001
Overall BW		y = -1.73(± 0.459) × 2 + 1871.12(± 40.393)	0.7478	*P* < 0.001
Overall BWG		y = -1.73(± 0.459)x^2^ + 1.09(± 9.589)x + 1831.56(± 40.478)	0.0697	*P* < 0.001
Overall FCE		y = -0.01(± 0.003)x + 0.66(± 0.012)	0.8120	*P* < 0.001

*BW* body weight; *BWG* body weight gain; *FCE* feed conversion efficiency; *FI* feed intake

### Carcass characteristics and visceral macromorphometry

Tables 5 and 6 show the effect of dietary incremental levels of MSM on carcass traits and weights and lengths of visceral organs of broilers, respectively, with their regression equations in Table 7. The results demonstrated that dietary inclusion of MSM linearly decreased SW (*P* < 0.001), HCW (*P* < 0.001), CCW (*P* < 0.001), dressing percentage (*P* < 0.001), and breast weight (*P* < 0.05) of birds. Also, dietary MSM linearly increased the weights of the liver (*P* < 0.01), proventriculus (*P* < 0.001), gizzard (*P* < 0.001), duodenum (*P* = 0.01), jejunum (*P* < 0.001), ileum (*P* < 0.001), caecum (*P* < 0.01), and colon (*P* < 0.01).

**Table 5:** Effect of dietary MSM inclusion on carcass traits (% of CCW, unless stated otherwise) of broilers

Parameter	Dietary inclusion of MSM (%)					SEM	*P*-value	
	0	5	10	15	20		Linear	Quadratic
Slaughter weight (SW) (g)	1902.19^a^	1770.94^b^	1711.88^b^	1559.06^c^	1171.25^d^	40.958	0.001	0.001
Hot carcass weight (HCW) (g)	1640.63a	1524.22^b^	1473.44^b^	1342.19^c^	998.61^d^	33.390	0.001	0.003
Cold carcass weight (CCW) (g)	1595.94^a^	1485.63^b^	1434.38^b^	1296.21^c^	982.28^d^	30.721	0.001	0.002
Dressing %	73.38^a^	72.58^a^	72.11^a^	70.31^b^	67.37^c^	0.538	0.001	0.008
Breast (%)	17.44^a^	15.09^ab^	16.46^ab^	14.39^b^	14.56^b^	0.988	0.048	0.672
Drumstick (%)	6.31	6.16	5.91	5.71	5.93	0.246	0.123	0.386
Thigh (%)	7.04	6.55	6.76	6.41	6.69	0.285	0.351	0.355
Wing (%)	4.62	4.72	4.84	4.68	4.76	0.164	0.623	0.604
Back length (cm)	21.54	20.54	19.41	21.15	22.66	2.248	0.683	0.343

*MSM* mucuna seed meal; *SEM* standard error of the mean

^a,b,c,d^ Means within the same row having different superscripts are considered significantly different

**Table 6:** Effect of dietary MSM inclusion on weights and lengths of visceral organs (% of HCW, unless stated otherwise) of broilers

Parameter	Dietary inclusion of MSM (%)					SEM	*P*-value	
	0	5	10	15	20		Linear	Quadratic
Liver weight (%)	2.34^b^	2.31^b^	2.26^b^	2.42^b^	2.80^a^	0.099	0.002	0.004
Spleen weight (%)	0.14	0.12	0.12	0.11	0.14	0.013	0.753	0.072
Proventriculus weight (%)	0.46^d^	0.52^ cd^	0.58^c^	0.73^b^	0.95^a^	0.040	0.001	0.009
Gizzard weight (%)	2.16^c^	2.24^bc^	2.24b^c^	2.25^b^	3.30^a^	0.087	0.001	0.001
Duodenum weight (%)	1.09^b^	1.17^b^	1.01^b^	1.20^b^	1.65^a^	0.135	0.010	0.034
Jejunum weight (%)	1.81^c^	1.96^c^	1.75^c^	2.27^b^	2.86^a^	0.082	0.001	0.001
Ileum weight (%)	1.51^b^	1.39^b^	1.39^b^	1.64^b^	2.44^a^	0.105	0.001	0.001
Caecum weight (%)	0.69^b^	0.72^b^	0.73^b^	0.76^b^	1.02^a^	0.052	0.002	0.012
Colon weight (%)	0.16^b^	0.17^b^	0.16^b^	0.17^b^	0.26^a^	0.024	0.009	0.074
Duodenum length (cm)	32.18	33.49	34.67	34.24	31.04	1.090	0.653	0.082
Jejunum length (cm)	75.91	80.58	78.10	83.29	82.28	2.460	0.054	0.690
Ileum length (cm)	84.19	82.92	82.64	83.89	87.79	2.885	0.363	0.265
Caecum length (cm)	21.19	21.92	20.19	20.06	20.50	0.872	0.245	0.753
Colon length (cm)	6.67	7.67	8.36	7.44	6.48	0.557	0.727	0.105

*MSM* mucuna seed meal; *SEM* standard error of the mean

^a,b,c,d^ Means within the same row having different superscripts are considered significantly different

**Table 7:** Regression equations of carcass characteristics and internal organs

Parameter	Regression equations	R^2^	*P*-value
Slaughter weight (SW)	y = -1.73(± 0.459)x^2^ + 1.20(± 9.569)x + 1871.12(± 40.393)	0.0702	*P* < 0.001
Hot carcass weight (HCW)	y = -1.53(± 0.380)x^2^ + 1.24(± 7.926)x + 1612.63(± 33.453)	0.0724	*P* < 0.01
Cold carcass weight (CCW)	y = -1.41(± 0.345)x^2^—0.098(± 7.201)x + 1571.64(± 30.395)	0.0679	*P* < 0.01
Dressing percentage	y = -0.02(± 0.006)x^2^ + 0.036(± 0.118)x + 73.20(± 0.499)	0.0683	*P* < 0.01
Breast weight	y = -0.22(± 0.222)x + 17.11(± 0.938)	0.1009	*P* < 0.05
Liver weight	y = 0.003(± 0.001)x^2^—0.04(± 0.022)x + 2.37(± 0.092)	0.1648	*P* < 0.01
Proventriculus weight	y = 0.001(± 0.0004)x^2^ + 0.0005(± 0.009)x + 0.47(± 0.037)	0.0561	*P* < 0.01
Gizzard weight	y = 0.005(± 0.001)x^2^—0.05(± 0.021)x + 2.23(± 0.087)	0.1891	*P* < 0.001
Duodenum weight	y = 0.003(± 0.001)x^2^—0.04(± 0.030)x + 1.15(± 0.127)	0.0980	*P* < 0.05
Jejunum weight	y = 0.005(± 0.001)x^2^—0.05(± 0.020)x + 1.88(± 0.084)	0.1783	*P* < 0.001
Ileum weight	y = 0.006(± 0.001)x^2^—0.08(± 0.023)x + 1.54(± 0.098)	0.2717	*P* < 0.001
Caecum weight	y = 0.001(± 0.001)x^2^—0.02(± 0.011)x + 0.72(± 0.049)	0.1132	*P* < 0.05
Colon weight	y = 0.01(± 0.005)x + 0.17(± 0.022)	0.1566	*P* < 0.01

### Haemato-biochemistry

Responses in haematology and serum biochemistry of broilers fed dietary graded levels of MSM is shown in Table 8, with their regression equations in Table 9. There were neither linear nor quadratic responses of all haematological parameters, except for heterophils (neutrophils) which quadratically increased (*P* < 0.05) with increasing dietary inclusion levels of MSM. Regarding serum biochemistry, there was a decrease in SDMA (linear: *P* = 0.001) and cholesterol (linear: *P* < 0.05) in contrast to the increase in alkaline phosphatase (quadratic: *P* < 0.05) as well as amylase (linear: *P* = 0.001) and lipase (linear: *P* = 0.001) activities in response to increasing dietary MSM levels.

**Table 8:** Effect of dietary inclusion of MSM on haematological parameters of broilers

Parameter	Dietary inclusion of MSM (%)					SEM		*P*-value
	0	5	10	15	20		Linear	Quadratic
*Haematology*								
Red blood cells (× 10^12^/L)	0.80	0.93	1.32	0.90	1.06	0.178	0.066	0.561
Haematocrit (L/L)	9.63	9.15	11.76	10.25	13.70	1.1632	0.095	0.570
Haemoglobin (g/dL)	10.35	10.24	9.94	11.08	10.79	0.272	0.628	0.920
White blood cells (× 10^9^/L)	11.39	9.35	9.14	11.60	11.94	2.056	0.071	0.378
Heterophils (× 10^9^/L)	3.25^ab^	2.23^ab^	1.63^b^	2.06^a^	3.97^a^	0.784	0.126	0.041
Lymphocytes (× 10^9^/L)	5.43	4.95	5.78	7.26	5.34	1.309	0.133	0.853
Monocytes (× 10^9^/L)	8.20	1.17	1.01	1.64	1.60	2.579	0.121	0.302
Eosinophils (× 10^9^/L)	0.94	0.79	0.67	0.65	1.18	0.146	0.121	0.075
Basophils (× 10^9^/L)	0.08	0.05	0.05	0.08	0.16	0.028	0.100	0.072
Platelet (K/µL)	92	70.47	60.29	149.50	131.56	21.64	0.938	0.321
*Serum biochemistry*								
Glucose (mg/dL)	146.44	146.19	142.19	146.69	146.69	6.194	0.959	0.693
SDMA (µg/dL)	21.13^ab^	22.00^a^	19.00^b^	18.00^b^	14.06^c^	0.892	0.001	0.029
Phosphate (mg/dL)	6.28	6.09	6.03	5.80	5.81	0.187	0.390	0.744
Calcium (mg/dL)	7.77	7.96	8.23	8.70	8.14	0.258	0.080	0.188
Total protein (g/dL)	3.04	3.14	3.30	3.08	3.18	0.114	0.557	0.367
Albumin (g/dL)	1.21	1.21	1.23	1.22	1.21	0.029	0.836	0.684
Globulin (g/dL)	1.85	1.93	2.05	1.98	1.97	0.078	0.241	0.197
Albumin/globulin	0.64	0.63	0.59	0.63	0.62	0.018	0.391	0.208
Alanine transaminase (U/L)	13.50	11.75	11.88	12.63	12.56	0.793	0.689	0.181
Alkaline phosphatase (U/L)	343.25^a^	321.50^a^	282.69^b^	370.69^a^	416.13^a^	33.786	0.073	0.05
Total bilirubin (mg/dL)	0.16	0.14	0.17	0.13	0.16	0.020	0.772	0.683
Cholesterol (mg/dL)	108.38^a^	110.69^a^	106.00^a^	106.25^a^	96.13^b^	4.378	0.040	0.223
Amylase (U/L)	300.06^b^	287.88^b^	427.06^b^	652.63^a^	743.25^a^	71.798	0.001	0.282
Lipase (U/L)	113.63^c^	146.00^c^	204.19^c^	317.19^b^	422.44^a^	32.183	0.001	0.096

*MSM* mucuna seed meal; *SDMA* symmetric dimethylarginine; *SEM* standard error of the mean

^a,b,c^ Means within the same row having different superscripts are considered significantly different

**Table 9:** Regression equations of haematological and serum biochemistry parameters

Parameter	Regression equations	R^2^	*P*-value
Heterophils	y = 0.33(± 0.016)x^2^ – 0.33(± 0.360)x + 2.577(± 1.692)	0.2479	*P* < 0.05
SDMA	y = -0.02(± 0.009)x^2^ + 0.07(± 0.199)x + 21.37(± 0.842)	0.0624	*P* < 0.05
Alkaline phosphatase	y = 0.75(± 0.358)x^2^ – 11.03(± 7.460)x + 345.18(± 31.448)	0.0973	*P* < 0.05
Cholesterol	y = -0.56(± 0.958)x + 108.43(± 4.042)	0.1054	*P* < 0.05
Amylase	y = 8.34(± 15.934)x + 273.66(± 67.256)	0.4452	*P* < 0.001
Lipase	y = 4.32(± 7.001418)x + 111.58(± 29.552)	0.6126	*P* < 0.001

*SDMA* symmetric dimethylarginine

### Meat quality

The meat quality of broilers fed diets supplemented with varying inclusion levels of MSM is shown in Table 10, with associated regression equations in Table 11. Regarding meat pH and colour at 45 min post slaughter, there were neither linear nor quadratic responses to dietary treatment (*P* > 0.05). However, dietary MSM linearly decreased meat pH at 24 h post slaughter (*P* < 0.05) as it linearly increased its cooking loss (*P* < 0.01), drip loss (*P* < 0.05) and shear force (*P* < 0.01).

**Table 10:** Effect of dietary MSC inclusion on meat quality of broilers

Parameter	Dietary inclusion of MSM (%)					SEM	*P*-value	
	0	5	10	15	20		Linear	Quadratic
pH_45 min_	6.11	6.12	6.13	6.11	6.11	0.033	0.899	0.603
Temperature_45 min_ (℃)	29.35	30.21	29.81	29.87	30.81	0.915	0.624	0.179
L*_45 min_	54.34	54.37	56.31	54.66	54.85	1.326	0.756	0.510
a*_45 min_	1.99	2.41	1.78	2.18	2.46	0.268	0.418	0.473
b*_45 min_	2.04	1.49	1.43	0.56	1.39	0.409	0.093	0.210
H*_45 min_ (˚)	0.82	0.49	0.64	0.48	0.46	0.138	0.088	0.263
C*_45 min_	2.90	3.01	2.45	2.44	3.05	0.324	0.789	0.206
pH_24 h_	6.17^ab^	6.20^a^	6.10^b^	6.16^ab^	6.08^b^	0.026	0.013	0.519
Temperature_24 h_ (℃)	10.88	10.46	8.59	9.81	10.14	0.722	0.359	0.098
L*_24 h_	52.93	52.85	53.96	52.66	53.29	1.179	0.884	0.822
a*_24 h_	3.62	3.74	3.45	3.49	3.91	0.291	0.699	0.391
b*_24 h_	3.39	2.42	2.41	1.92	2.64	0.441	0.157	0.082
H*_24 h_ (˚)	0.75	0.57	0.58	0.51	0.57	0.089	0.147	0.226
C*_24 h_	5.02	4.53	4.13	4.17	4.92	0.346	0.609	0.266
Cooking loss (%)	24.02^b^	25.63^b^	26.29^b^	27.05^b^	30.99^a^	1.186	0.002	0.287
Drip loss (%)	8.12^b^	9.23^ab^	9.02^ab^	10.46^a^	9.43^ab^	0.552	0.035	0.218
WHC (%)	84.64	85.60	82.51	82.48	80.98	2.825	0.239	0.857
Shear force (*N*)	6.65^b^	7.13^b^	8.68^b^	9.19^b^	12.85^a^	1.109	0.002	0.199

*MSM* mucuna seed meal; *SEM* standard error of the mean; *WHC* water holding capacity

^a,b^ Means within the same row having different superscripts are considered significantly different. a*—redness, b*—yellowness, C*—chroma, H*—hue angle, L*—lightness

**Table 11:** Regression equations of meat quality parameters

Parameter	Regression equations	R^2^	*P*-value
pH_24 h_	y = -0.001(± 0.006)x + 6.18(± 0.026)	0.1548	*P* < 0.05
Cooking loss	y = 0.04(± 0.262)x + 24.40(± 1.104)	0.3100	*P* < 0.01
Drip loss	y = 0.23(± 0.124)x + 8.11(± 0.525)	0.1102	*P* < 0.05
Shear force	y = 0.02(± 0.012)x + 6.77(± 1.027)	0.3121	*P* < 0.01

## Discussion

This study aimed at determining the optimum dietary inclusion level of MSM partially substituting SBM in broiler diets in terms of chicken performance, health, and meat quality. Revealing a decrease in FI, BW, BWG and FCE in response to incremental dietary MSM levels, our data mimicked those of Akinmutimi and Okwu (2006) and Akure et al. (2021). In terms of FI, the data however suggested good palatability of the legume supplement from 5 to 15% dietary inclusion levels, beyond which the observed sharp decrease in this parameter, as well as in BW, BWG and FCE, depicted detrimental effects of MSM-bound ANFs. Indeed, MSM naturally contains high levels of L-DOPA, tannins, phytic acid, oxalate, and other phytochemicals (Sarmiento-Franco et al. 2019; Mthana et al. 2022) that compromise feed palatability, appetite, BWG and FCE in broilers (Del Carmen et al. 1999; Akinmutimi and Okwu 2006; Omidiwura et al. 2016). Also, the presence of a high content of fibre in MSM would have induced poor feed digestibility (Akure et al. 2021) that would account for the observed decrease in chicken BW, BWG and FCE from as low dietary inclusion level of MSM as 5%. Further, the decrease in BW and BWG was mirrored in the depressed carcass characteristics in corroboration with earlier observations (Tuleun and Igba 2008). The decreased meat yield suggests MSM-derived ANF-induced poor absorption and utilization of nutrients otherwise necessary for muscle tissue synthesis.

Also, the observed increased weights of essential organs particularly the liver and digestive tract corroborate a recent study by Zungu et al. (2023). However, in contrast to these researchers, more metabolic-digestive tissues were negatively affected by MSM in the current study. This is probably due to variations in the age of birds between the two studies. Zungu et al. (2023) used rather mature birds at finisher phase that already had metabolic and digestive organs more adapted to processing diets with ANFs and fibre compared to the younger birds used in the current investigation. Nonetheless, our data reflect hypertrophic effects on the liver and digestive organs of MSM-derived L-DOPA and other phytochemicals (Osei and Dei 1998; Oloruntola et al. 2018) as well as dietary fibre (JøRgensen et al. 1996; Jha and Mishra 2021). Aside L-DOPA and other ANFs, indeed MSM contains a high level of fibre (CF: 97 to 193 g/kg DM) (Belewu and Olajide 2010; Pathania et al. 2020) with a high content of non-starch polysaccharides (Musthafa et al. 2018) that would induce antinutritional effects on the birds. Thus, the observed hypertrophic effects of MSM on the liver and digestive organs were not unexpected as this study employed rather high dietary inclusion levels of the native legume unlike previous investigations wherein lower (4 to 15 g/kg) and hence less detrimental inclusion levels were tested (Oloruntola et al. 2018; Ayodele et al. 2021).

Also, the data on haemato-biochemistry further support the supposed mechanism of deleterious effects of MSM-bound ANFs in broilers fed high dietary levels of the indigenous legume. Indeed, the observed linear increase in serum amylase and lipase activities suggest MSM-induced pancreatic damage (acute pancreatitis) to the birds. Mostly secreted by the pancreas, both amylase and lipase are respectively required for the digestion of starch and triglycerides (Pieper-Bigelow et al. 1990). However, when the pancreas is acutely damaged, abnormal intracellular activation of trypsinogen to trypsin occurs, *inter alia*, leading to pancreatic cell autolysis and increased leakage of amylase and lipase into the circulation, with the serum activity of these enzymes consequently elevated 3 times beyond the normal range (Elfar et al. 2007; Regasa et al. 2022; Beiriger et al. 2023). Our data thus suggest the presence of some chemical substance(s) in MSM that evidently induced acute pancreatitis as evidenced by increased serum amylase and lipase activities in birds fed high dietary levels of MSM. We speculated that the putative mechanism may involve L-DOPA in MSM inhibiting pancreatic insulin secretion leading to hyperglycaemia (Ericson et al. 1977) and diabetes-like inflammation of the pancreas accompanied by elevated serum amylase and lipase (Lazarus and Volk 1961; Hardt et al. 2003). Alternatively, L-DOPA might have induced recurrent pancreatitis (Jitkritsadakul et al. 2013), if not the relatively high content of L-arginine in mucuna seeds (Hernández-Orihuela et al. 2022) having induced acute pancreatitis elicited through inflammation, oxidative stress, apoptosis and increased serum activities of amylase and lipase (Mores et al. 2023; Ono et al. 2023; Xia et al. 2023). These results agree with those of Ojha et al. (2014) who found elevated intestinal amylase and lipase in fish fingerlings fed diets supplemented with the ethanolic extract of *Mucuna pruriens* seeds. Despite the observed diet-induced elevations, our serum amylase and lipase values mimicked those of Alabi et al. (2024) obtained from the same strain of Ross 308 broilers (amylase: 391.4 – 601.5 U/L; lipase: 189.6 – 415.6 U/L).

On the other hand, the quadratic response of chicken blood heterophils may be associated with the phytochemicals in MSM. Indeed, mucuna seeds are naturally endowed with a variety of phytochemicals including tannins, phytic acid, oxalate, L-DOPA and others (Sarmiento-Franco et al. 2019) that are known to impart beneficial effects at low and antinutritional effects at high dietary concentrations (Nath et al. 2022). It would therefore appear that the phytochemicals were beneficial to the chickens at 5 and 10% inclusion levels of MSM but began to induce toxicity to the birds at 15 and particularly 20% inclusion levels. L-DOPA particularly would induce oxidative stress-mediated toxicity at high dietary concentrations (Takasaki and Kawakishi 1997). This would therefore explain the observed decreased blood heterophils at low in contrast to increased counts of these cells at high inclusion levels of MSM. Since elevated levels of heterophils are associated with inflammation (Reeves et al. 1993; Belluzzi et al. 2000; De Silva et al. 2010), the elevated response of these immune cells in chickens fed the 20% MSM-supplemented diet suggests that these birds suffered enormous amount of MSM’s L-DOPA-induced inflammation and possibly oxidative stress. This argument is further supported by the quadratic response in serum alkaline phosphatase with incremental dietary levels of MSM. Whilst serum activity of this enzyme is an important clinical indicator of liver function and bone metabolism (Petronijević et al. 2008; Kazmi et al. 2022), it is also a biomarker for inflammation (Spector et al. 1997; Abramowitz et al. 2010) with its serum levels positively associated with inflammatory markers including C‐reactive protein (CRP) levels and leukocyte counts (Webber et al. 2010).

Notwithstanding, broiler consumption of MSM-containing diets did not induce any deleterious effects on kidney function as shown by decreasing SDMA responses to dietary increasing levels of the legume seed meal. In this regard, increased SDMA levels are correlated with acute kidney damage (Kielstein et al. 2006) with SDMA values < 15 µg/dL considered normal and those > 20 µg/dL abnormally indicative of kidney injury (IDEXX 2023). Though it tested inclusion levels (2.5% to 10%) lower than in the current study, a previous study found no effects of feeding diets supplemented with MSM on SDMA responses in broilers (Zungu et al. 2023).

Otherwise, the observation of linearly decreasing cholesterol concentrations in response to incremental dietary MSM levels intriguingly corroborated previous findings from broilers fed MSM (Jayaweera et al. 2007). Indeed, the seeds of mucuna plant contain a plethora of bioactive compounds including saponins, phenolics and flavonoids with hypocholesterolemic properties (Shi et al. 2004; Hashem and Kaviani 2010; Raksha et al. 2014). Our finding is therefore of great interest considering the worrying reports of global incident cases of cardiovascular disease (CVD) having increased by 77.12% from 1990 (31.31 million) to 2019 (55.45 million), with CVD-related deaths having risen by 53.81% (to 18.56 million) during this period, particularly in developing countries of southern Africa (Li et al. 2023). Hence our observation of hypocholesterolemic effects of MSM in broilers has positive implications for human health in terms of producing meat with health-promoting properties and de-linking chicken-derived cholesterol with CVD (Jeong et al. 2018).

On meat quality, the linear decrease in meat ultimate pH at 24 h post-slaughter with increasing dietary MSM levels may be due to post-mortem muscle anaerobic glycolysis required to convert muscle glycogen to lactic acid (Khatun et al. 2020). This may be an indication of the high carbohydrate content of MSM enhancing the glycolytic potential (i.e., the molar sum of glucose, glucose-6-phosphate, glycogen, and lactate) of broiler meat. Indeed, despite the presence of ANFs and high fibre content, both of which would be envisaged to limit carbohydrate digestion and thus decrease muscle anaerobic glycolysis (Gemede and Ratta 2018; Soetan 2018; Thakur et al. 2019), mucuna seeds contain a high concentration of carbohydrates (59.5 – 64.9%) (Baby et al. 2023) relative to SBM (35 – 40%) (Choct et al. 2010). Otherwise, our meat ultimate pH values were slightly lower than those of Mthana et al. (2022) but within the range previously observed in broilers fed alternative protein sources (Gunya et al. 2019) and normal range (5.5 – 6.5) recommended for chicken meat (Hertanto et al. 2018).

Lastly, the linearly increased meat cooking and drip losses in response to dietary MSM are evidently related to the diet-induced decrease in meat pH and WHC. Indeed, low chicken meat pH has previously been associated with low WHC, which leads to cooking and drip losses, due to low pH decreasing muscle proteins’ ability to bind to water, which causes myofibrils shrinkage (den Hertog-Meischke et al. 1997). Otherwise, the diet-induced increase in meat cooking loss could also be due to smaller muscle fibres in broilers fed MSM-containing diets. Smaller muscle fibres tend to lose too much water during dripping and cooking (Ryu and Kim 2006). Further, the increase in meat cooking loss might account for the observed MSM-induced increase in meat shear force. During cooking, shrinkage of smaller muscle fibres is higher relative to that of larger muscle fibres and lowers meat tenderness due to meat dryness (Rao et al. 2022). Otherwise, the observed increase in meat cooking loss corroborates reports by Dahouda et al. (2009) and Mthana et al. (2022) in their respective studies with guinea fowls and broilers fed MSM containing diets.

## Conclusion

Our results showed that dietary inclusion of high levels of MSM decreased overall performance and carcass characteristics of broilers whilst increasing the weights of their metabolic and digestive organs. The optimum dietary inclusion level of MSM was found to be 10%. Therefore, there is a need for a pre-treatment strategy such as with enzymes to quench the detrimental effects of ANFs in the legume to allow its optimal utilization as an alternative to SBM in broiler diets.

## Data Availability

The data used is available upon request from the corresponding authors.
